# Amino acid changes in PB2 and HA affect the growth of a recombinant influenza virus expressing a fluorescent reporter protein

**DOI:** 10.1038/srep19933

**Published:** 2016-02-05

**Authors:** Hiroaki Katsura, Satoshi Fukuyama, Shinji Watanabe, Makoto Ozawa, Gabriele Neumann, Yoshihiro Kawaoka

**Affiliations:** 1 Division of Virology, Department of Microbiology and Immunology, Institute of Medical Science, University of Tokyo, Tokyo, Japan; 2 Exploratory Research for Advanced Technology, Infection-Induced Host Responses Project, Japan Science and Technology Agency, Saitama, Japan; 3 Laboratory of Veterinary Microbiology, Department of Veterinary Sciences, University of Miyazaki, Miyazaki, Japan; 4 Laboratory of Animal Hygiene, Joint Faculty of Veterinary Medicine, Kagoshima University, Kagoshima, Japan; 5 Transboundary Animal Distance Center, Joint Faculty of Veterinary Medicine, Kagoshima University, Kagoshima, Japan; 6 Department of Pathobiological Sciences, School of Veterinary Medicine, University of Wisconsin-Madison, Madison, WI, USA; 7 Department of Special Pathogens, International Research Center for Infectious Diseases, Institute of Medical Science, University of Tokyo, Tokyo, Japan

## Abstract

Influenza viruses that express reporter proteins are useful tools, but are often attenuated. Recently, we found that an influenza virus encoding the Venus fluorescent protein acquired two mutations in its PB2 and HA proteins upon mouse adaptation. Here, we demonstrate that the enhanced viral replication and virulence in mice of this Venus-expressing influenza virus are primarily conferred by the PB2-E712D mutation, with only a minor contribution by the HA-T380A mutation.

Recombinant influenza A viruses that express reporter proteins are useful tools for studying the dynamics of influenza virus infection *in vivo*^1^,^2^. Building on the strategy of Manicassamy *et al*.^3^, we recently generated an influenza A/Puerto Rico/8/34 (PR8, H1N1) virus expressing the Venus reporter protein from a fusion construct with the viral NS1 protein^4^. This virus (referred to as wild-type [WT]-Venus-PR8) was attenuated *in vitro* and *in vivo*, but serial passages in mice resulted in a mouse-adapted variant (MA-Venus-PR8) whose replicative ability and virulence in mice were similar to those of wild-type PR8 (WT-PR8) virus^4^. MA-Venus-PR8 differs from WT-Venus-PR8 by two amino acid changes: one in the viral polymerase subunit PB2 (PB2-E712D) and the other in the hemagglutinin (HA) surface glycoprotein (HA-T380A; H1 HA numbering)^4^. Here, we generated single-gene reassortants and assessed the contribution of these mutations to viral growth and pathogenicity to mice.

## Results and Discussion

**Figure 1:**
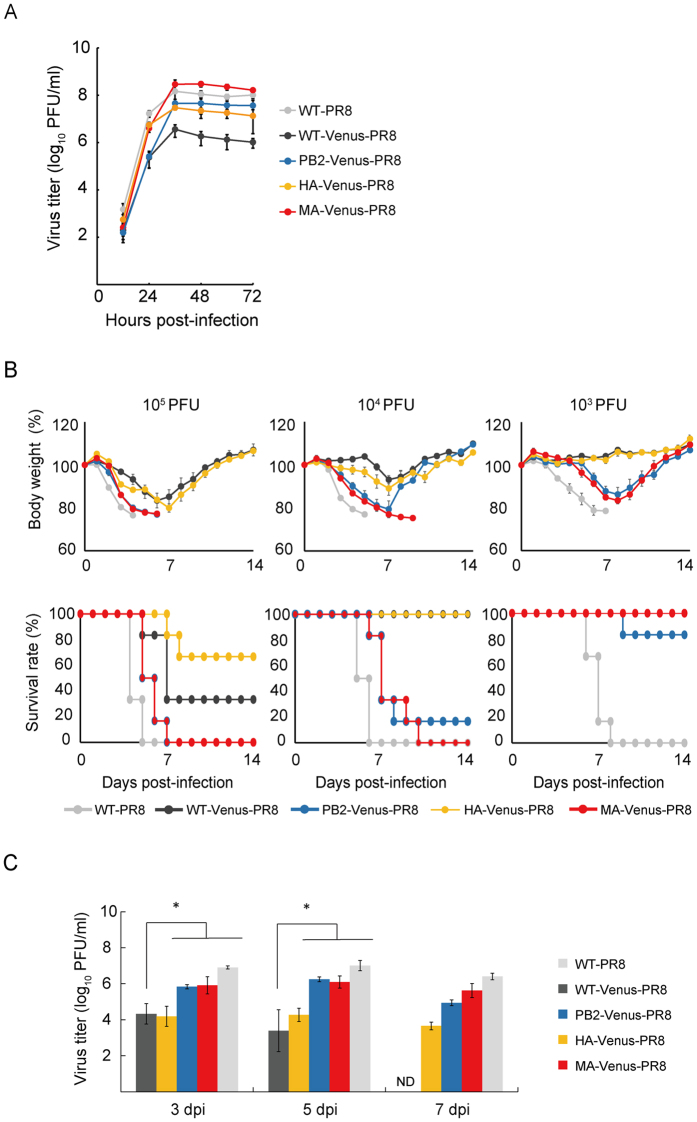
Viral replication in MDCK cells and virulence in mice. (**A**) Virus replication in MDCK cells. Results are expressed as the mean titer (log_10_ PFU/ml) ± standard deviation. (**B**) Assessment of virulence in mice. Body weights are shown as the mean ± standard error. (**C**) Assessment of virus titers in the lungs of infected mice. Results are expressed as the mean of the titer (log_10_ PFU/g) ± standard deviation. Statistical significance was calculated by using the Tukey-Kramer method. Asterisks indicate significant differences from titers in mice infected with WT-Venus-PR8 (*P* < 0.05). ND: Not detected (detection limit, 5 PFU/lung).

To assess the contributions of PB2-E712D and HA-T380A to the increased replicative ability of MA-Venus-PR8, we used reverse genetics^5^ to generate two single-gene reassortants that possessed the PB2 or HA viral RNA (vRNA) of MA-Venus-PR8 and the remaining vRNA segments from WT-Venus-PR8 (referred to as PB2-Venus-PR8 and HA-Venus-PR8, respectively). We infected Madin-Darby canine kidney (MDCK) cells at a multiplicity of infection (MOI) of 0.001 and determined viral titers in supernatants by using plaque assays (Fig. 1A). As reported previously^4^, MA-Venus-PR8 grew to significantly higher titers than WT-Venus-PR8. The PB2 and HA vRNAs of MA-Venus-PR8 each increased the growth properties of WT-Venus-PR8 virus, although the virus titers did not reach that of MA-Venus-PR8. These data indicate that the PB2-E712D and HA-T380A mutations both contributed to increased virus replication in MDCK cells.

In mice infected with 10^3^–10^5^ plaque-forming units (PFU) of viruses, MA-Venus-PR8 was more virulent than WT-Venus-PR8, as reported previously^4^ (Fig. 1B). The increased virulence of MA-Venus-PR8 compared with WT-Venus-PR8 was primarily conferred by the MA-Venus PB2 vRNA, whereas HA-Venus-PR8 was similar in its virulence to WT-Venus-PR8. These findings were consistent with virus titers in the lungs of mice infected with 10^3^ PFU of each virus (Fig. 1C). Hence, the PB2-E712D substitution is primarily responsible for the increased virulence of MA-Venus-PR8 relative to WT-Venus-PR8 in mice.

**Figure 2:**
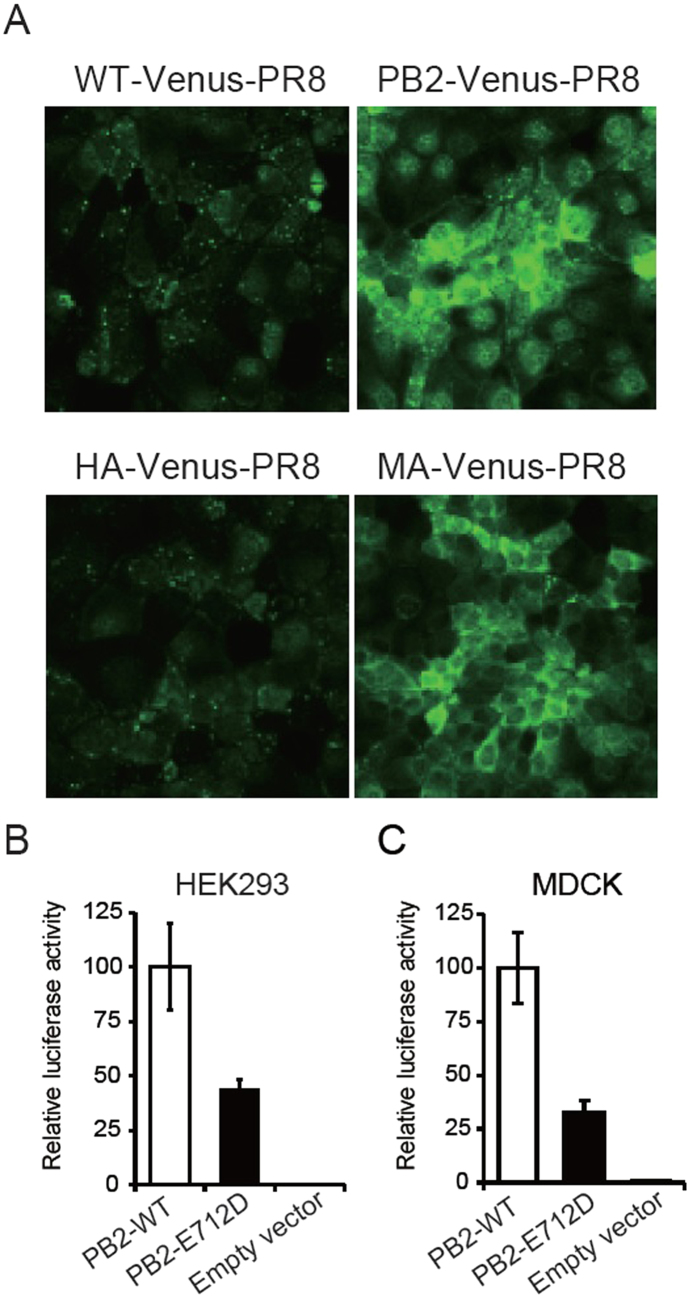
The PB2-E712D substitution augments the expression of Venus in infected cells but does not enhance the polymerase activity in minireplicon assays. (**A**) Venus expression in infected MDCK cells. MDCK cells were infected with each virus at an MOI of 1 and observed 12 h later by confocal microscopy. (**B**) Polymerase activity in minireplicon assays in human HEK293 cells. Cells were transfected with plasmids encoding the PB1, PA, NP, and wild-type or mutant PB2 proteins, with a plasmid for the expression of the virus-like RNA encoding the firefly luciferase gene under the control of the human RNA polymerase I promoter, and with a control plasmid encoding *Renilla* luciferase. Luciferase activity was measured 48 h later. (**C**) Polymerase activity in minireplicon assays in canine MDCK cells. Cells were transfected with plasmids encoding the PB1, PA, NP, and wild-type or mutant PB2 proteins, with a plasmid for the expression of the virus-like RNA encoding the firefly luciferase gene under the control of the canine RNA polymerase I promoter, and with a control plasmid encoding *Renilla* luciferase. Luciferase activity was measured 48 h later.

To demonstrate that the PB2-E712D mutation increased the Venus expression levels, we infected MDCK cells with the indicated viruses at an MOI of 1 and performed confocal microscopy 12 h later (Fig. 2A). As expected, the levels of the NS1-Venus fusion protein were higher in cells infected with MA-Venus-PR8 or PB2-Venus-PR8 than in those infected with WT-Venus-PR8 or HA-Venus-PR8 (Fig. 2A).

Collectively, our data indicate that the PB2-E712D substitution is primarily responsible for the increased replicative ability, Venus expression, and virulence in mice of MA-Venus-PR8 virus. To assess whether the PB2-E712D mutation directly affects the viral polymerase activity, we performed a minireplicon assay in human HEK293 cells^6^,^7^. Unexpectedly, the polymerase activity of PB2-E712D was lower than that of the parental PB2 (Fig. 2B). Similar results were obtained with canine MDCK cells^8^ (Fig. 2C). In the context of a minireplicon that measures viral replication and transcription, the PB2-E712D mutation is thus attenuating; in contrast, this mutation enhances viral growth in the context of replicating virus. These findings indicate that the PB2 protein functions not only in viral replication/transcription, but performs additional roles in the viral life cycle.

**Figure 3:**
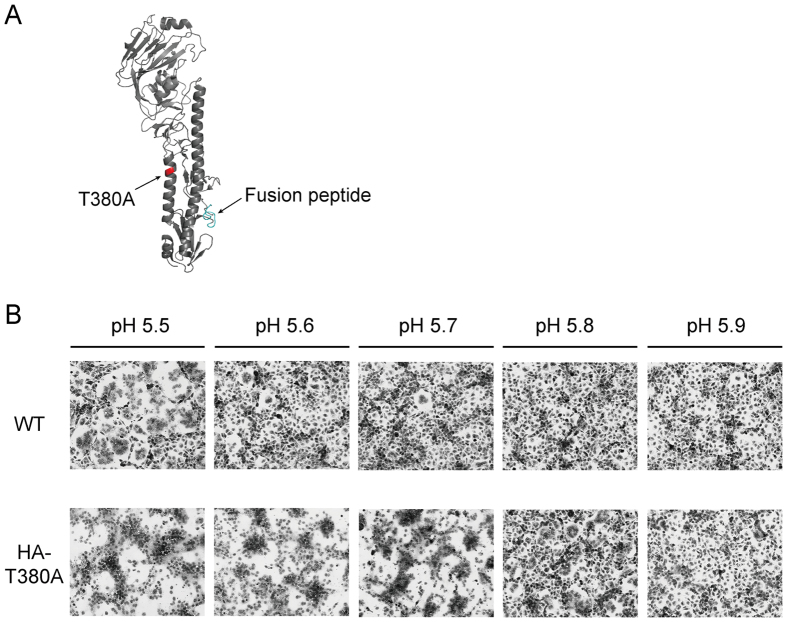
The HA-T380A substitution affects the pH threshold for membrane fusion of HA. (**A**) The position of the HA-T380A substitution (red) was mapped on the PR8 HA three-dimensional structure^9^. The three-dimensional structure of PR8 HA was obtained from the Protein Data Bank (PDB ID: 1RU7). The fusion peptide of HA is shown in cyan. The image was generated by using Pymol software. (**B**) The pH threshold of HA-mediated membrane fusion. Representative results of two independent experiments are shown.

The HA vRNA of MA-Venus-PR8 did not significantly increase the virulence of WT-Venus-PR8 in mice; however, HA-Venus-PR8 virus grew more efficiently in MDCK cells than WT-Venus-PR8 (Fig. 1A), suggesting a contribution of the HA-T380A mutation to, at least, virus replication in cultured cells. Because the HA-T380A substitution is located on an α-helix in the HA2 subunit^9^ (Fig. 3A), we evaluated its effect on HA membrane-fusion activity by using a polykaryon formation assay^10^. The wild-type HA had a threshold for membrane fusion of pH 5.5, whereas the threshold for HA-T380A was pH 5.8 (Fig. 3B), leading to the conformational change in HA at an earlier stage of endosome maturation during influenza virus entry^11^. Changes in the pH threshold for membrane fusion may affect HA thermostability^12^, an effect that we did not observe at 50 °C (data not shown).

In conclusion, the increased replicative ability, Venus expression, and virulence in mice of MA-Venus-PR8 are primarily brought about by the PB2-E712D mutation, with a minor additional contribution by HA-T380A. The glutamic acid residue at PB2-712 is highly conserved among all influenza A viruses; our search of the Influenza Research Database (www.fludb.org) did not uncover a single isolate with an aspartic acid residue at PB2-712. Interestingly, the PB2-E712D mutation does not increase viral replication and transcription in minireplicon assays perhaps because it affects PB2 binding to importins, which are essential for protein import into the nucleus. The PB2 interaction with importin is mediated by the PB2 residues at positions 701^13^ and, to a lesser extent, 714^14^. In fact, the PB2-D701N and PB2-S714R mutations facilitate adaptation of avian influenza viruses to mammals by affecting the PB2 interaction with importin, resulting in better exposure of the PB2 nuclear localization signal^13^,^14^. A mutation at position 712 (which is located in the three-dimensional structure of the viral polymerase complex next to the residue at position 714^15^) may affect the PB2 interaction with importins, potentially reducing the virus’s replicative ability. On the other hand, PB2 is known to interact with RIG-I^16^, and may also interact with other cellular factors that play roles in innate immune responses^17^,^18^ or in the assembly and budding of new virions^18^. We speculate that the PB2-E712D mutation may affect these steps, resulting in the higher virulence of the PB2-E712D mutant virus *in vivo*.

The NS1 protein is incorporated into progeny virions^19^. NS1 functions as an antagonist of host immune responses by interfering with innate immune pathways such as the RIG-I/IPS-1 signaling pathway, resulting in the inhibition of interferon secretion^20^. Although we have not examined differences in NS1 protein incorporation between WT-Venus-PR8 and mouse-adapted viruses, it is possible that PB2-Venus-PR8 and MA-Venus-PR8 incorporated more NS1 protein because NS1-Venus expression levels were higher in cells infected with these viruses. Since the virion-incorporated NS1 protein could function soon after infection, PB2-Venus-PR8 and MA-Venus-PR8 might suppress the host innate immune responses more efficiently than WT-Venus-PR8, resulting in the higher proliferative ability and virulence of the mouse-adapted viruses.

Our hypothesis that PB2 has additional, as-yet uncharacterized roles in the viral life cycle is supported by two other recent studies^21^,^22^. Alternatively, higher NS1 virion-incorporation levels may increase virulence. Although the mechanism is not currently understood, PB2-E712D and HA-T380A (in this study), as well as other mutations found in the MA-Venus-H5N1^23^ virus, are useful for generating recombinant fluorescent influenza viruses that replicate efficiently in cultured cells and mice.

## Methods

### Cells and viruses

Madin-Darby canine kidney (MDCK) cells were maintained in minimum essential medium (MEM) containing 5% of newborn calf serum. Human embryonic kidney 293 (HEK293) cells were maintained in Dulbecco’s modified Eagle medium supplemented with 10% fetal calf serum (FCS). PR8 and each Venus-PR8 mutant were generated by using reverse genetics^5^ and were propagated in MDCK cells at 37 °C for 48 h in MEM containing L-(tosylamido-2-phenyl) ethyl chloromethyl ketone (TPCK)-treated trypsin (0.8 μg/ml) and 0.3% bovine serum albumin (BSA).

### Growth kinetics of each virus

MDCK cells were infected with each virus at an MOI of 0.001. Supernatants were collected every 12 h and viral titers in the supernatants were determined by means of plaque assay in MDCK cells.

### Pathogenicity and replication of viruses in mice

Six-week-old female C57BL/6 mice were intranasally infected with 50 μl of 10^3^, 10^4^ or 10^5^ PFU of each virus. Six mice per group were monitored for survival and body weight changes for 14 days after infection. Three mice per group were infected with 10^3^ PFU of each virus and euthanized on the indicated days. Their lungs were collected to determine viral titers by means of plaque assay in MDCK cells. All animal experiments were performed in accordance with the University of Tokyo’s Regulations for Animal Care and Use, which were approved by the Animal Experiment Committee of the Institute of Medical Science, the University of Tokyo (approval number PA 10–13).

### Minigenome assay

A minigenome assay based on the dual-luciferase system was performed as described previously^6^,^7^,^8^. Briefly, HEK293 cells were transfected with viral protein expression plasmids for PB1, PA, NP and wild-type PB2 or PB2-E712D, with a plasmid expressing a reporter vRNA encoding the firefly luciferase gene under the control of the human RNA polymerase I promoter [pPolI/NP(0)Fluc(0)], and pRL-null (Promega), which expresses *Renilla* luciferase, as a transfection control. The luciferase activity in the transfected HEK293 cells were measured by using Dual-Glo luciferase assay system (Promega) at 48 h posttransfection. Polymerase activity was calculated by standardization of the firefly lucifearase activity to the *Renilla* luciferase activity. For the minigenome assay in canine MDCK cells, we used a plasmid expressing a reporter vRNA encoding the firefly luciferase gene under the control of the canine RNA polymerase I promoter^8^.

### Polykaryon formation assay

Ploykaryon formation assay was performed as described previously^10^ with modification. HEK293 cells were infected with wild-type PR8 or PR8 possessing the HA-T380A mutation in DMEM containing 10% FCS at an MOI of 10. At 18 h post-infection, cells were washed with MEM containing 0.3% BSA and treated with TPCK-treated trypsin (1 μg/ml) in MEM containing 0.3% BSA for 15 min at 37 °C to cleave the HA on the cell surface into HA1 and HA2. Trypsin was inactivated by washing the cells with DMEM containing 10% FCS. To initiate polykaryon formation, cells were exposed to low-pH buffer (145 mM NaCl, 20 mM sodium citrate (pH 6.0–5.4)) for 2 min at 37 °C. Then the low-pH buffer was replaced with DMEM containing 10% FCS and the cells were incubated for 2 h at 37 °C. The cells were then fixed with methanol and stained with Giemsa’s solution.

## Additional Information

**How to cite this article**: Katsura, H. *et al*. Amino acid changes in PB2 and HA affect the growth of a recombinant influenza virus expressing a fluorescent reporter protein. *Sci. Rep*. **6**, 19933; doi: 10.1038/srep19933 (2016).
